# Late Working Life Patterns in Sweden

**DOI:** 10.1093/geroni/igab046.148

**Published:** 2021-12-17

**Authors:** Gülin Öylü, Susanne Kelfve, Andreas Motel-Klingebiel

**Affiliations:** 1 LinkÃ¶ping University, Norrköping, Ostergotlands Lan, Sweden; 2 Linköping University, Norrkoping, Ostergotlands Lan, Sweden; 3 Linköping University, Linkoping University, Ostergotlands Lan, Sweden

## Abstract

Late working life patterns differ across different social groups and birth cohorts. The mechanisms of these participation differences and role of working life policies can be understood better by using additional working life indicators and historical perspective. This paper investigates how late working life patterns of different age, gender, education groups and birth cohorts are structured in Sweden using participation, employment type, employment break and exit trajectories of different groups. Using Swedish National Registry Data, employment trajectories of the age groups of 60-68 of the birth cohorts 1930, 1935, 1940, 1945 and 1950 are followed. Results show that for all birth cohorts, participation is higher for younger age groups, men and higher educated; leaving the working life before 61 is more common among primary educated; changing employment type in late working life is observed more among higher educated men and usage of sickness compensation is higher among primary educated and women. However, the peak age that individuals exit, and experience employment breaks differs over the years. In addition, although higher educated individuals have lower shares in usage of unemployment and sickness compensation for all birth cohorts, the structure of benefits usage of the other education and gender groups change over the years. Overall, results give important insights how changes in working life policies affect working life patterns of different groups over the years.

